# Comparison of antibody responses of heterologous and homologous Covid-19 booster vaccination: an observational study

**DOI:** 10.3389/fimmu.2024.1448408

**Published:** 2024-11-13

**Authors:** Nayab Batool Rizvi, Maryam Bibi, Muhmmad Zeeshan Rana, Sehrish Zaffar, Hassam Farooq

**Affiliations:** ^1^ Center for Clinical and Nutritional Chemistry, School of Chemistry, University of the Punjab, Lahore, Pakistan; ^2^ Chemical Pathology Department, Combined Military Hospital (CMH), Lahore, Pakistan; ^3^ Pharmacology Department, Combined Military Hospital (CMH) Medical College & Institute of Dentistry, Lahore, Pakistan

**Keywords:** COVID-19, virus, vaccine, booster, homologous model

## Abstract

**Objective:**

Pakistan has been seriously affected by the COVID-19 pandemic, with numerous waves of infection. Using different vaccine and booster doses was a key component to control and combat this pandemic. This study aims to monitor the heterologous and homologous booster vaccination doses that generate immune responses in healthy adults after 9 months of vaccination.

**Methods:**

In this cross-sectional, observational study a total of 173 samples were collected. Participants from both genders (Male and Female) between the ages of 18 to 25 years were enrolled for the study. Participants who had booster shots of homologous Sinopharm BBIBP CorV and heterologous Pfizer-BioNTech vaccines were included only, with the use of a Roche Cobas-e601 analyzer, the antibody titers in the blood serum were quantified by the ECLIA method. IBM SPSS 22 was utilized for descriptive statistical analysis and P< 0.05 was considered significant.

**Results:**

In this study the IgG antibody levels were measured against the full length of receptor binding domain (RBD) of the spike (S) protein. The mean antibody titer in the Pfizer group was 9764 ± 10976 U/mL and 5762 ± 4302 U/mL in the Sinopharm group. The Mean IgG antibody levels of the Pfizer-vaccinated group were significantly higher than the Sinopharm-vaccinated group (P=0.000, each). Comparing the Sinopharm BBIBP CorV booster dosage to the Pfizer booster, Pfizer BNT162b2demonstrated a stronger immune response. However, there were no immunological gender-specific significant differences. The administration of a third dosage of Pfizer BNT162b2 after two doses of BBIBP CorV

**Conclusion:**

The administration of a third dosage of Pfizer BNT162b2 after two doses of BBIBP-CorV is recommended to boost the humoral immune response in the general population while there was no gender-specific difference observed. More effectiveness can be attained by administering additional doses due to the antibody decay.

## Introduction

The Coronavirus Disease 2019 (COVID-19) has emerged as one of the greatest challenges to humanity since World War II. The economies of various countries have been severely affected due to this pandemic, significantly the economy of Developing countries like Pakistan (1). Pakistan faced six waves of the COVID-19 pandemic from June 2020 to April 2023. According to the published reports more than 1,580,327 infected cases and 30,654 deaths have been confirmed in Pakistan due to the COVID-19 pandemic till April 16, 2023 (2).

In the absence of targeted therapeutics and drugs, innate and acquired immunity were the primary defenders against SARS-CoV-2. The entry of the foreign elements (Viruses) triggers the initiation of an immune response with the establishment of immune memory. The neutralizing antibodies present in the serum that persist for an extended duration offer long-lasting protection (3). Hence induction and reinforcement of the humoral response through vaccination is considered as the fundamental strategy for effectively controlling and managing COVID-19 infection. Within the humoral response, the production of antibodies ensures the elimination of viruses and the relative prevention of infection (4).

The government of Pakistan launched a massive immunization campaign by establishing various vaccine deployment centers in all districts of the country. A wide range of vaccines were deployed, offering diverse mechanisms of action comprising mRNA vaccines, namely Moderna mRNA-1273 and Pfizer BNT162b2; inactivated vaccines, including Sinopharm and CoronaVac Sinovac; and non-replicating viral vector-based vaccines, such as AstraZeneca, CanSino/PakVac, and Sputnik V (1).

Despite the initial stages of vaccination, the risk of the pandemic persisted. Several factors contributed to reinfection, including vaccine hesitancy, the mutation of the spike protein, and the decay of COVID-19 neutralizing antibodies. In light of these factors, researchers advised the administration of booster shots to enhance protection against the deadly virus. The booster shots for the COVID-19 vaccine were also deployed by the Government of Pakistan, however only 48.04 million vaccinees received the third booster dose (5).

A booster dose refers to an extra administration of the vaccine. This booster dose may consist of the same product used in the initial series (homologous) or a different product (heterologous) (6).

The Pfizer BioNTech (BNT162b2) is an mRNA-based vaccine that has been modified to encode a complete spike protein. In phase I and II clinical trials, the analysis of immune responses revealed the induction of Th1-skewed activity in the majority of participants. Elevated amounts of T-cell growth factor (IL-2), lymphokine-12 (IL-12), and type II interferon (IFN) were found in the assays, which provided proof of this. Th1-skewed activity refers to a preferential activation and dominance of Th1 cells, which are a specific subset of CD4+ T cells. It is worth noting that T-cell immune responses have a longer duration and contribute to the establishment of long-term immunity. The phase III clinical trials demonstrated a vaccine efficacy of 95%, particularly among the older and more vulnerable population. Importantly, no instances of serious toxicity were observed during these trials (7). A non-significant difference between vaccinated females and males were observed, when anti-S1 and anti-RBD antibody levels were analyzed in the subjects who were injected with mRNA vaccine for SARS-CoV-2 (8). Inoculation of supplementary or booster doses of mRNA vaccines may present a viable approach for substantially augmenting the immunogenicity elicited by standalone inactivated virus vaccines. Consequently, this strategy holds the potential to confer protection against both established and emerging variants, thus bolstering overall efficacy (9, 10).

Beijing Bio-institute of Biological Products (BBIBP)/Sinopharm tested beta-propiolactone inactivated whole virus as a COVID-19 vaccine candidate with an alum adjuvant. Clinical trials carried out during the initial stage of COVID-19 demonstrated that BBIBP-CorV exhibited sufficient effectiveness in minimizing new infections and COVID-19-associated deaths caused by SARS-CoV-2. The expected protective effectiveness was 78.89% (95% CI 65.79%, 86.97%), while vaccine effectiveness, considering person-years of follow-up, was 78.07% (95% CI 64.82%, 86.33%). Notably, there were no significant differences in vaccine efficacy between males and females, with point estimates of 78.4% and 75.6%, respectively. These findings support the safety and effectiveness of BBIBP-CorV in a real-world deployment (11).

The Sinovac vaccine is believed to operate using a similar approach as Sinopharm, but mRNA vaccines are considered better surrogates according to studies. For those who received the classical vaccines Sinopharm/Sinovac or those facing challenges with their immunity, a booster dose is recommended (12, 13). The heterologous regimen of mRNA vaccine with initial two doses of inactivated vaccine was found more effective against SARS CoV-2 new variants (14). The deployment of heterologous booster shots for protection against Coronavirus, enhances immune response by increasing both the functionality and quantity of T follicular helper (Tfh) cells. This promotes B-cell activation and antibody production. The mechanism of presenting varied antigens, activates innate immune pathway and enhances CD4 T-cell responses. This mechanism results in higher number of antibody producing cells and enhanced cross protection against different variants of virus, leading to more robust and strong immune response (15).

This prospective observational study aims to monitor heterologous and homologous vaccine booster doses induced IgG antibody response of two different vaccines BBIBP CorV Sinopharm and Pfizer BNT162b2 in healthy adults and to estimate gender-specific immune response differences. The antibody IgG levels were measured after 9 months of administration of a booster dose to monitor antibody decay. Monitoring post-vaccination immune response and antibody production among vaccinees after valuable outcomes will aid in the selection of the most efficacious vaccine in the future and a more targeted approach can be implemented to control pandemics.

## Methods

### Patient and public involvement statement

This observational study was conducted at the University of the Punjab, Lahore, Pakistan, between February 2022 and March 2023. The research was approved by ethical review committees of the University of the Punjab, Lahore, the University of Lahore, Combined Military Hospital, Lahore, and CMH Lahore Medical College, Lahore Pakistan (File no. 2265-TRG).

### Inclusion criteria

The study included participants of both genders (male and female) aged between 18 and 25 years. The participants underwent a full vaccination regimen, nine months prior to onset of this study, i.e. received first two doses of an inactivated whole virus vaccine (either Sinopharm or Sinovac) and also received booster doses of Pfizer and Sinopharm, respectively. A total of 173 samples (of which 95 females and 78 males) of both genders were collected from four places: the University of Punjab, the University of Lahore, CMH Lahore Medical College, and the Combined Military Hospital, Lahore, Pakistan. The ratio of male to female was 54.9% (females) and 45.1% (males) respectively. Using vaccination cards provided by Pakistan’s National Database and Registration Authority (NADRA), the participants’ age and immunization status were confirmed. Informed written and verbal consent was obtained from all participants or their legal guardians. The confidentiality of the data was assured to the respondents.

### Exclusion criteria

Participants with a history of active or previous infection with COVID-19, recent or incomplete vaccination, or any documented medical condition were excluded from the study, after taking a detailed medical history of last three years. Additionally, individuals with comorbidity or undergoing treatment with antibiotics, corticosteroids, or immunosuppressants were also excluded from the study population.

### Sample collection

Informed consent, both verbal and written, was obtained from all participants, with a strong emphasis on maintaining the confidentiality of their data. 3 ml of venous blood was collected from each participant, which was carefully collected using aseptic procedures by trained lab staff and added to serum collection tubes. The cellular residue was separated from the serum, and the resulting serum was stored in capped vials at a temperature of -80°C until further analysis. Approximately 1.5 ml aliquots were utilized for the analysis.

To analyze the immune response of anti-SARS-CoV-2 RBD antibodies, the Roche Diagnostics test kit entitled (Elecsys Anti-SARS-CoV-2) was employed. The measurement and quantitative analysis of immunoglobulin G (IgG) were carried out using the electrochemiluminescence immunoassay (ECLIA) double antigen sandwich method on a fully automated Hitachi Cobas e601 analyzer. The assay kit’s measuring range spanned from 0.40 to 250 U/ml, with a concentration threshold of >0.80 U/ml. In case exceeding the kit’s range, dilutions of 1:10, 1:100, or 1:400 were applied using the manufacturer-provided diluents (Roche diluents universal 2 for Elecsys Cobas e-analyzers).

Before sample analysis, the Roche Cobas e601 analyzer underwent calibration and quality control procedures to ensure optimal performance. Each test kit had a capacity of 200 samples. The Elecsys Anti-SARS-CoV-2 S kit (Roche Diagnostics) exhibited a manufacturer’s claimed level of precision of 99.98% and sensitivity of 98.8% (16).

### Statistical analysis

Version 22 of IBM SPSS was used for the descriptive statistical study. Categorical variables were presented as frequencies and percentages. Numerical variables were presented as standard deviations. Independent t-test was employed to assess the difference in antibody levels between male and female participants, of both vaccines. Univariate regression analysis was applied to evaluate the impact of gender and vaccine type on mean antibody titer levels. A significance level of p<0.05 was considered statistically significant for this study.

## Results

The mean age of the participants was 22 years. Of the 173 participants, 79 received booster doses of Pfizer-BNT162b2, while 94 received a booster of Sinopharm BBIBP-CorV, as shown in **Table 1**.

**Table 1:** Participants in Pfizer and Sinopharm vaccine group (*n* = 173).

Participants		Vaccine		Total
		Pfizer	Sinopharm	
Female	Count	42	53	95
	% within Vaccine	53.2%	56.4%	54.9%
Male	Count	37	41	78
	% within Vaccine	46.8%	43.6%	45.1%
Total	Count	79	94	173
	% within Vaccine	100.0%	100.0%	100.0%

**Figure 1:**
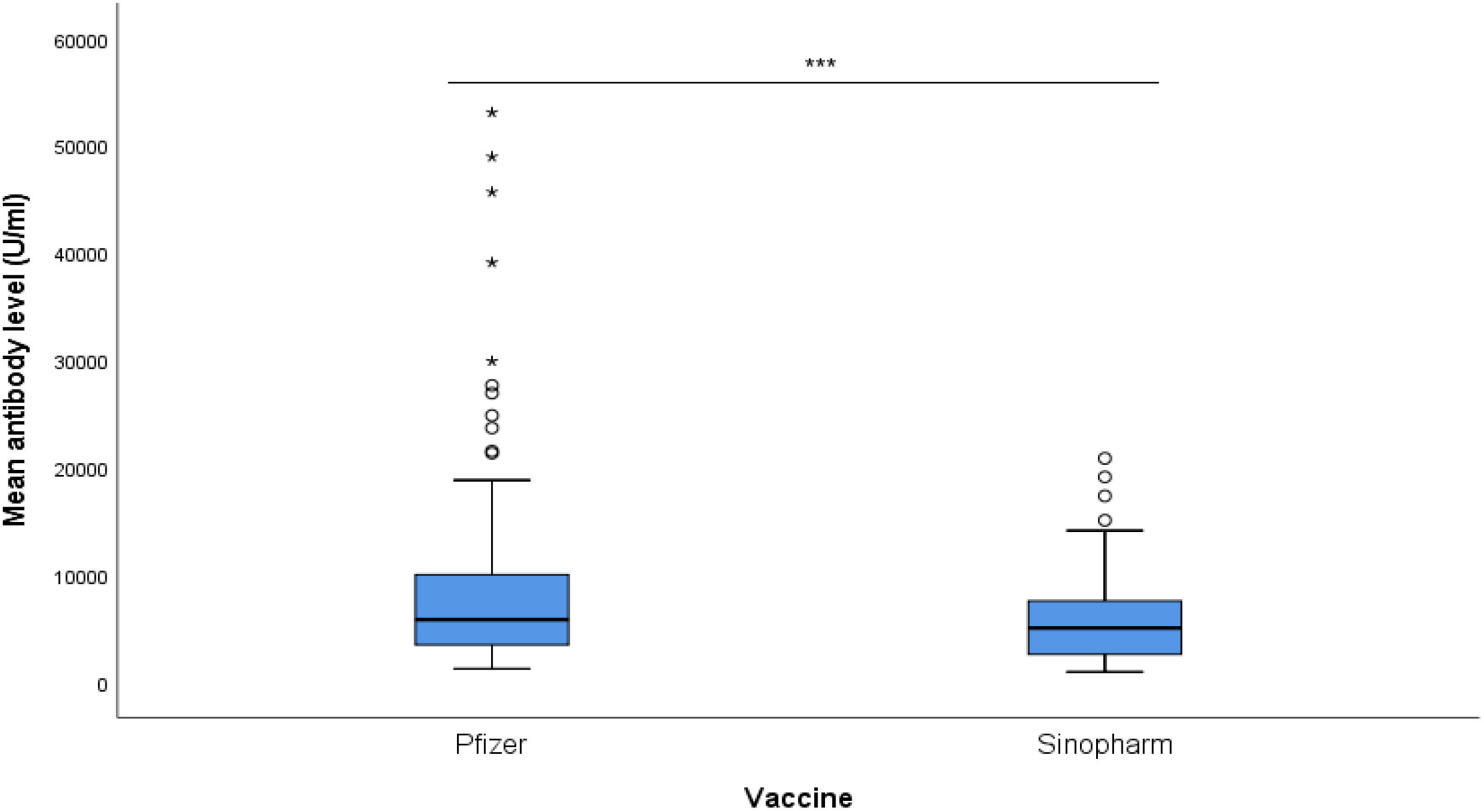
Mean antibody level (U/ml) of Pfizer and Sinopharm vaccine groups, illustrated in Box plot (n=173). *** - p<0.001. No statistically significant difference was observed between the mean antibody levels of males and females, receiving either Pfizer or Sinopharm booster dose. Gender-wise comparisons of mean antibody levels in Pfizer and Sinopharm groups are visualized in **Table 2** and **Figure 2**. "asterisk" these symbols indicates levels of significant differences, greater the number of asterisks greater will be the significant differences.

The Pfizer-BNT162b2 group had a mean antibody level of 9764 ± 10976 U/ml, while the Sinopharm BBIBP-CoV group had a mean antibody level of 5762 ± 4302 U/ml, as illustrated in **Figure 1**. With a p-value of 0.001, the difference between the two groups was found to be statistically significant.

**Table 2:** Gender-wise comparison of mean antibody level (U/ml) in Pfizer and Sinopharm vaccine group (n=173).

Vaccine	Gender	Number of participants	Mean antibody level (U/ml)	Std. Deviation	Std. Error of Mean	p-value
Pfizer	Female	42	7895.98	5981.01	922.89	0.107
	Male	37	11884.62	14546.98	2391.51	
Sinopharm	Female	53	5877.60	4543.96	624.16	0.770
	Male	41	5613.56	4019.82	627.79	

**Figure 2:**
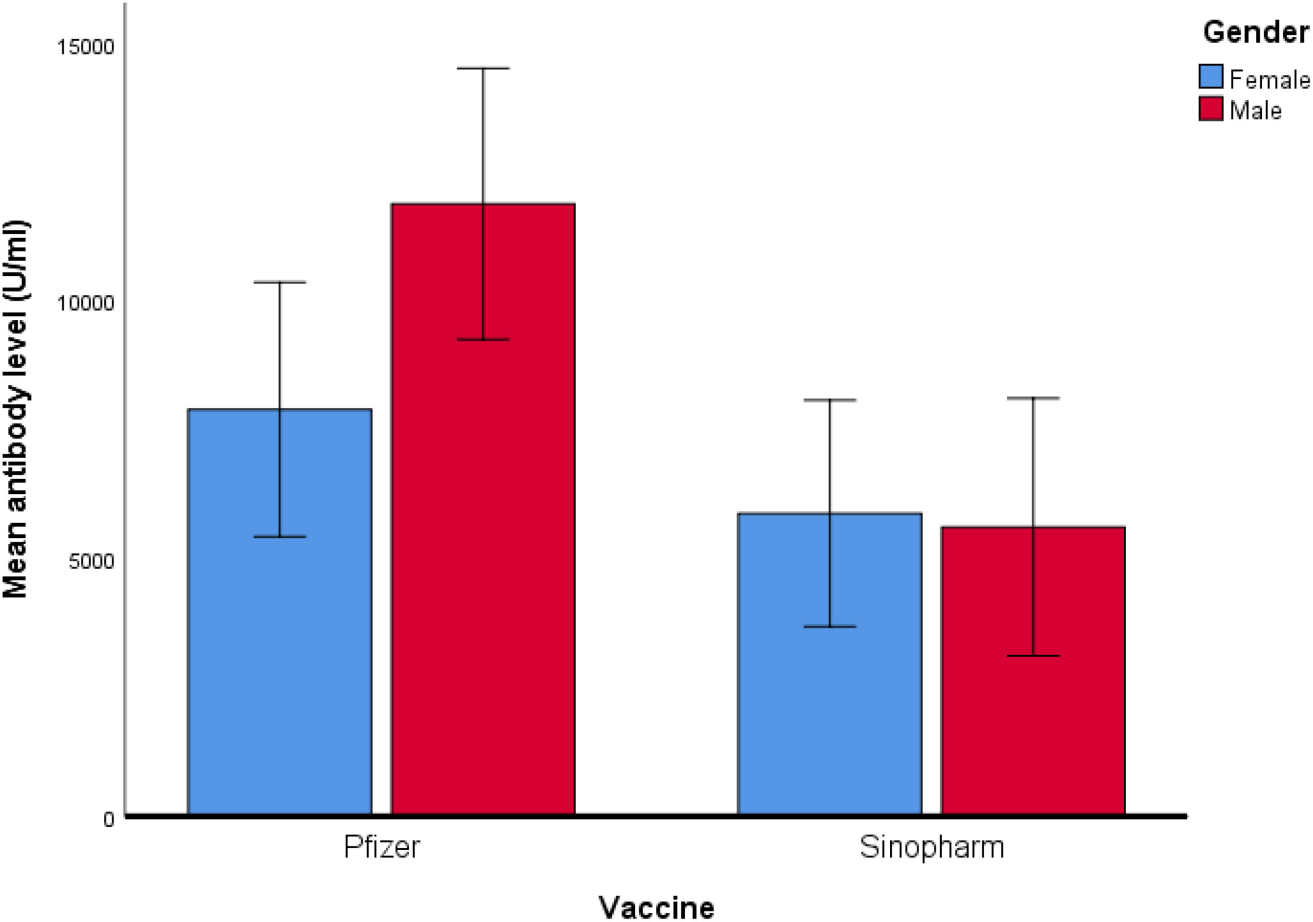
Comparison of mean antibody level (U/ml) in Pfizer and Sinopharm vaccine group (n=173). Log10 algorithm was applied to transform the data, followed by univariate linear regression to observe the impact of gender and different types of vaccines on mean antibody level. **Table 3A** reveals a significantly higher mean antibody level in the Pfizer group, as compared to the Sinopharm group. However, the difference in mean antibody levels between males and females was not statistically significant, as detailed in **Table 3B**.

**Table 3A:** Effect of vaccine type on mean IgG antibody level by linear regression.

Vaccine type	Coefficient Beta	Standard Error	Partial Eta Squared	Observed Power	Significance
Intercept	3.644	.037	.982	1.000	.000
Pfizer	.152	.055	.042	.777	.007
Sinopharm	*0^a^	.	.	.	.

•*This parameter is set to zero because it is redundant.

•Computed using alpha = 0.05.

^a^indicates that the parameter is set to zero as the reference category (Sinopharm), against which other vaccine types (e.g., “Pfizer”) are compared.

**Table 3B:** Effect of gender on mean IgG antibody level by linear regression.

Gender	Coefficient Beta	Standard Error	Partial Eta Squared	Observed Power	Significance
Intercept	3.713	.038	.982	1.000	.000
Male	-1.119E-5	.057	.000	.050	1.000
Female	0^a^	.	.	.	.

•This parameter is set to zero because it is redundant.

•Computed using alpha = 0.05

^a^indicates that the parameter is set to zero as the reference category (Sinopharm), against which other vaccine types (e.g., “Pfizer”) are compared.

## Discussion

This observational study was undertaken to compare heterologous Pfizer and homologous Sinopharm (booster doses) of the COVID-19 vaccinations, when Omicron variant of SARS-COV-2 was predominant in Pakistan (17). The samples of both genders (Male & Female) for antibody IgG analysis were collected after 9 months post-Covid-booster dosage. To the best of our knowledge, it is the first study conducted in Pakistan on the effectiveness and comparison of booster doses of COVID-19 vaccination. Our findings demonstrate that both heterologous and homologous vaccine booster doses generated anti-SARS-CoV-2 RBD antibodies, which play a crucial role in mitigating COVID-19. However, antibody responses were different. It was observed in other findings that receiving a third dose of the vaccine during both the Delta- and Omicron-predominant periods was found to be extremely effective at reducing the number of COVID-19-related hospitalizations. Reduction in severe COVID-19-related cases is associated to a timely administration of the booster doses (18).

As the production of antibodies ensures the elimination of viruses and prevents infection. This investigation determines that deployment of a heterologous vaccine regimen consisting of Pfizer BNT162b2 with two initial doses of Sinopharm induces a more effective antibody response when compared to a homologous vaccination comprising Sinopharm. These findings emphasize the advantage of combining different vaccine formulations to enhance immunogenicity. In previous studies, it was observed that a BNT162b2 booster elicited a greater immune response after two ChAdOx1 priming doses than after three doses of ChAdOx1. BNT162b2 was recommended by Keskin et al. as a booster following two CoronaVac priming doses (19). It was also noticed in a national study conducted on subjects aged 60 and above that those who received a third dose of the BNT162b2 vaccine had a much lower incidence of confirmed COVID-19 and severe disease (20). The delivery of the third dose mRNA vaccine was also supported by various clinical trials. Our results are in line with the studies showing an increase in antibody levels following a third dose of the COVID-19 vaccine BNT162b2 (21, 22).

However, in our previous published study (23), the immunity developed in same age group by two doses of Pfizer BNT162b2 remained superior (Male mean: 15,899.71& Female mean: 9401.01) to immune response produced by heterologous administration of Pfizer BNT162b2 booster dose after an initial two doses of inactivated virus vaccine regimen (Male mean: 11884.62& Female mean: 7895.98) measured in this study. The performance of Pfizer BNT162b2 as a third dose demonstrated its effectiveness greater when compared with a homologous regimen of inactivated booster doses (**Table 2**). While the comparison between the mean of antibody levels of just two doses of inactivated vaccine measured in our previous study (Male mean: 5301.054& Female mean: 5024.72) and those measured in this study with inactivated booster dose have minimal differences which indicates least effectiveness of inactivated whole virus vaccine as booster dose than mRNA i.e. Pfizer BNT162b2 vaccine. The homologous vaccination regimen of Pfizer BNT 162b2 (three doses of mRNA vaccine) could be a better option than heterologous regimen in order to develop more durable and stronger immune response. It was observed that when serum-neutralizing antibodies were analyzed after the administration of the initial two doses (findings of our previous study) and after the administration of a third inactivated vaccine dose (findings of our current study) the IgG antibodies demonstrated minimum variance. A possible factor of the least efficacy of inactivated whole virus vaccines might be due to the decline of antibodies with time. To investigate the factors contributing to decline of the immune response elicited by inactivated virus vaccines requires rigorous and extensive research endeavors. However, when comparing inactivated vaccine with mRNA vaccines in recent studies it was reported that inactivated vaccines as a booster resulted notably lower frequency of adverse effects when compared to mRNA vaccines. There is limited data available in which adverse effects of inactivated vaccine booster dosage are reported (9, 24, 25).

While monitoring neutralizing activities of vaccines, Sinopharm BBIBP-CorV showed least neutralization activity against both Delta and Omicron variants. A homologous booster dose improved neutralizing response, but in some cases it still lose its activity against Omicron variant, demonstrating low immunogenicity when compared to mRNA vaccine doses. While Sinopharm vaccine resulted in significant escape from vaccine-induced immune response by Omicron sub-variant (26), studies using mouse models suggested that mRNA vaccines, specially the Wuhan strain (WT) receptor binding domain, produce efficient neutralizing antibodies against other various sub-variants, however, their efficacy against Omicron is minimal. Interestingly, an mRNA vaccine for Omicron variant generates high concentration of antibodies but it is least effective for other variants. In contrast, hybrid vaccines provide greater protection against all COVID-19 sub-variants and highlights its importance for booster strategies in enhancing immune responses and managing variant escape (27).

In studies, it was reported that females generally display strong innate and acquired immunogenecity than males. Which leads to produce higher antibody response. Key hormones such as estrogen and testosterone are linked with higher concentration of antibodies in females and lower concentration in males respectively. Moreover genetic factors play crucial role: as female X chromosomes harbor additional immune related genes when compared with Y chromosomes in males. The sex related immunogenecity is also dependant on age. The older female demonstrated better efficacy than males although adverse reactions don’t necessarily reduce with age (28). However, in this study antibodies generated by mRNA booster doses in males were found significantly greater than females while in Sinopharm group there was a non-significant difference observed in male and female induced antibodies after administration of boosters. This pattern was also observed in our previous published study on same age group sample (23).

Many previous studies demonstrated that BNT162b2 is highly effective in preventing severe SARS-CoV-2 infection and mortality, particularly in those with declining immunity after primary immunization, and that booster doses of vaccination are probably required to limit SARS-CoV-2 transmission. The outcome of this study is consistent with the findings of various previous studies (29, 30) as antibody levels produced by Pfizer BNT162b2 as a booster dose demonstrated favorable outcomes while evaluation. To address the concern of wanning of immunity, a fourth vaccine dose is recommended, this strategy is already implemented in countries such as Israel, the United Kingdom, and the United States of America. The fourth dose was found to be more effective than the third dose (25).

One limitation of this study is its small sample size, while another concern is the absence of analysis for cellular immune response induced by the vaccines as this observation is entirely based on antibody response. Therefore, future research campaigns must address the important factors responsible for antibody decay. It is crucial to conduct large-scale population-based research to ensure perfect outcomes, with the surveillance of cellular immune responses.

## Conclusion

The Pfizer BNT162b2 booster dose immunization generated a significantly stronger immune response when compared to the booster dose of inactivated whole virus vaccine booster i.e. Sinopharm BBIBP-CorV. Hence, the Pfizer BNT162b2 booster dosage significantly raised antibody levels, indicating a vaccination approach that would offer superior defense against different SARS-COV-2 subtypes. Gender-Specific differences were non-significant, indicating that gender had no impact on vaccine effectiveness. These findings support the advice of Pfizer BNT162b2 (mRNA vaccine) booster dose administration since it provides promising protection. An additional booster dose of vaccines can develop a more durable immune response due to the decay of antibodies.

### Future perspectives

This study will be helpful for several health authorities for the deployment of COVID-19 vaccines and their booster doses which provide higher immune response, in the future. The study will facilitate subjects who have vaccine-induced lower immune response to select suitable booster doses. Future studies may focus on finding the reason for the decline of antibodies even after the administration of booster doses.

## Data Availability

The original contributions presented in the study are included in the article/supplementary material. Further inquiries can be directed to the corresponding author.
